# Clinical consequences of asbestos-related diffuse pleural thickening: A review

**DOI:** 10.1186/1745-6673-3-20

**Published:** 2008-09-08

**Authors:** Susan E Miles, Alessandra Sandrini, Anthony R Johnson, Deborah H Yates

**Affiliations:** 1 Dust Diseases Board Research & Education Unit, Sydney, NSW, Australia; 2 Department of Thoracic Medicine, St Vincent's Hospital, Darlinghurst, Sydney, NSW, Australia

## Abstract

Asbestos-related diffuse pleural thickening (DPT), or extensive fibrosis of the visceral pleura secondary to asbestos exposure, is increasingly common due to the large number of workers previously exposed to asbestos. It may coexist with asbestos related pleural plaques but has a distinctly different pathology. The pathogenesis of this condition as distinct from pleural plaques is gradually becoming understood. Generation of reactive oxygen and nitrogen species, profibrotic cytokines and growth factors in response to asbestos is likely to play a role in the formation of a fibrinous intrapleural matrix. Benign asbestos related pleural effusions commonly antedate the development of diffuse pleural thickening. Environmental as well as occupational exposure to asbestos may also result in pleural fibrosis, particularly in geographic areas with naturally occurring asbestiform soil minerals. Pleural disorders may also occur after household exposure. High resolution computed tomography (CT) is more sensitive and specific than chest radiography for the diagnosis of diffuse pleural thickening, and several classification systems for asbestos-related disorders have been devised. Magnetic resonance imaging and fluorodeoxyglucose positron emission tomography (PET) scanning may be useful in distinguishing between DPT and malignant mesothelioma. DPT may be associated with symptoms such as dyspnoea and chest pain. It causes a restrictive defect on lung function and may rarely result in respiratory failure and death. Treatment is primarily supportive.

## Introduction

Millions of people worldwide have been exposed to asbestos. The commonest manifestation of asbestos exposure is pleural disease, including pleural plaques and diffuse pleural thickening (DPT). Malignant mesothelioma of the pleura and DPT are less common than plaques, both conditions are likely to become more common in the future[1]. The overall prevalence of pleural disease including DPT is increasing due to the large number of workers who were exposed and the long latency of the disorder[2,3]. The Worker's Compensation Dust Diseases Board of New South Wales acknowledges an increase in DPT cases from 65 cases in 2002 to 133 cases in 2006. This review is primarily aimed at clinicians. It summarises available information on diffuse pleural thickening (DPT), contrasting it with other types of pleural disease, discusses potential pathogenetic mechanisms, and summarises available evidence regarding its clinical consequences.

**Figure 1:**
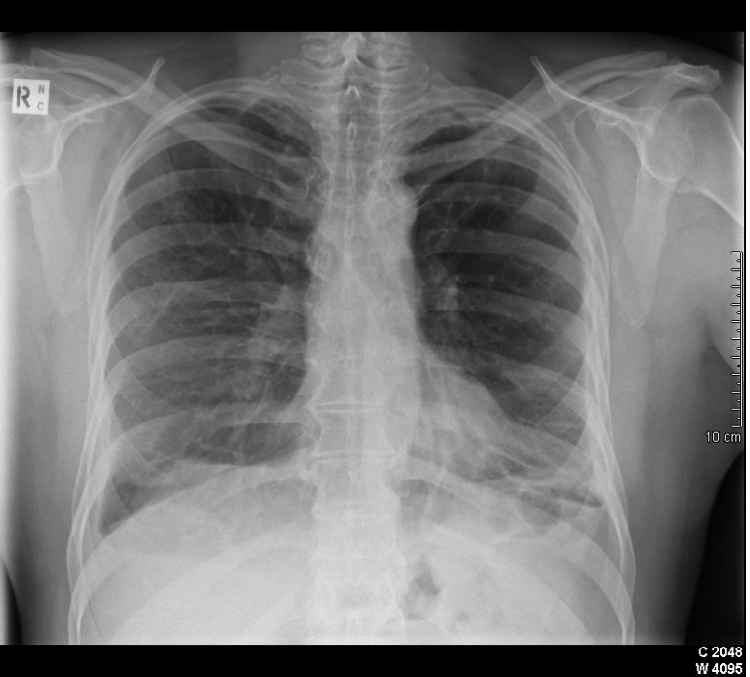
Postero-anterior chest radiograph demonstrating asbestos-related diffuse pleural thickening.

**Figure 2:**
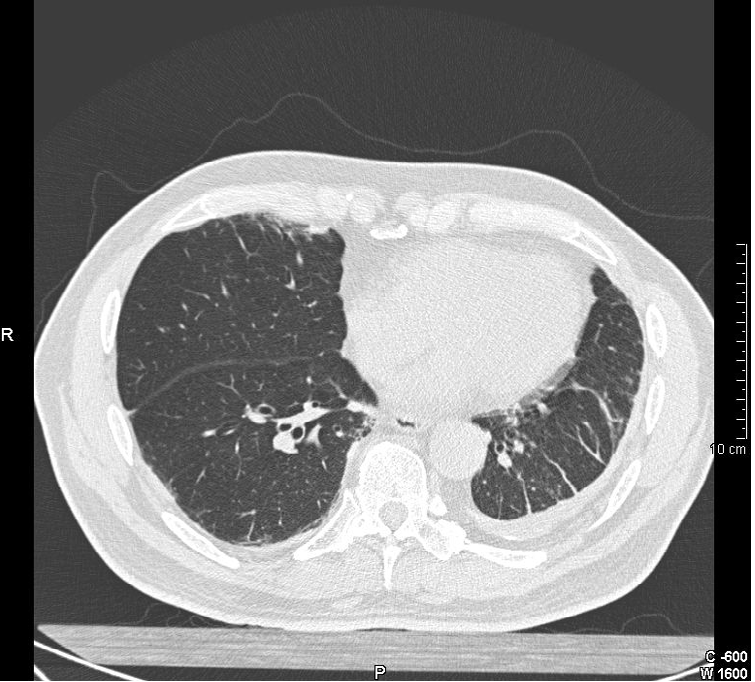
**Computed tomography (CT) scan of the thorax demonstrating asbestos-related diffuse pleural thickening.** Note the "crow's feet" or parenchymal bands which are clearly seen on the left, and the overall reduction in lung volume.

**Figure 3:**
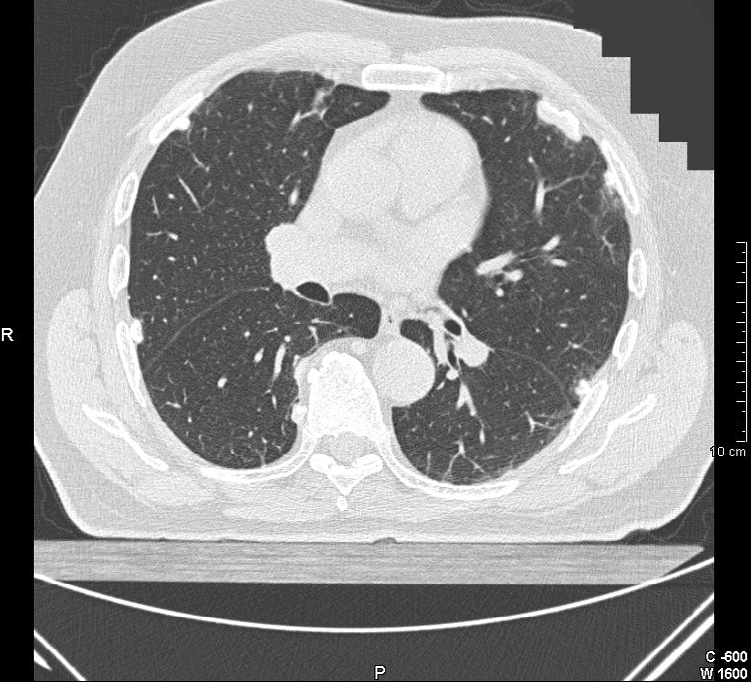
CT scan of the thorax demonstrating circumscribed calcified bilateral pleural plaques.

A link between pleural disease and asbestos exposure was first recognized in the 1930s [4] but it was not until the 1960s that a distinction between diffuse pleural thickening and pleural plaques was made[5]. Asbestos-related DPT refers to extensive fibrosis of the visceral rather than the parietal pleura, with adherence to the parietal pleura and obliteration of the pleural space (Figures 1 &2) [6,7]. In contrast, the parietal pleura is primarily involved in pleural plaques (Figure 3). DPT has unique radiographic features and significant symptomatic and functional consequences for affected patients[6]. It may cause exertional dyspnoea and has been associated with chest pain and in very rare cases with respiratory failure and death due to lung "constriction". Benign asbestos-related pleural effusions are believed to antedate the majority of cases of diffuse pleural thickening and to contribute towards disease progression. DPT may coexist with pleural plaques but has a distinctly different pathology, natural history and prognosis. Treatment is largely limited to supportive and symptomatic care, although rare case reports in the past have documented pleurectomy to be effective in a few progressive cases [8].

## Epidemiology

The prevalence of DPT is difficult to adequately document as this disorder is asymptomatic in its earliest stages. Prospective studies of asbestos workers have shown DPT to occur in between 5 – 13.5% of workers between 3–34 years following first asbestos contact [2,9]. In one large study of asbestos exposed insulators, where 58.2% of workers had pleural disease, DPT was rare compared with pleural plaques (5.5% vs 52.5%.[9]. The number of patients with DPT assessed for disablement benefit in the UK increased from 380 from April 2002 to 415 in 2004 (; last accessed on 27 July 2007) and is likely to be an underestimate of the true prevalence. Lower numbers of cases reported prior to this (150 in 1991) are likely to partly reflect changes in the method of collecting statistical information as well as changes in diagnostic criteria, as cases of unilateral DPT have only more recently been included for compensation.

In New South Wales, Australia, the total number of DPT cases notified to the Surveillance of Australian Workplace Based Respiratory Events of NSW (SABRE NSW) Scheme until 2005 was 503, reaching a prevalence of 74.3 cases per million. The number of new cases notified to the Scheme in 2006 was 120 for DPT, 143 for mesothelioma and 240 for pleural plaques alone, although these figures also are likely to be underestimates [10].

Prevalence of DPT increases in a population from the time of first asbestos exposure [11], partly because of disease progression but also because calcification occurs, which allows easier detection. The latency (or the time between exposure and first diagnosis of disease) is variable. DPT can develop within a year from exposure to asbestos, usually following a benign asbestos related pleural effusion, but may also take 15–20 years or more to be diagnosed. This contrasts with the documented latency for the development of pleural plaques and asbestosis, which is generally longer at between 20–30 years, and malignant mesothelioma, which may have an even longer latency period of 40 years or more[3,11].

Asbestos-related pleural disease is well documented to occur after environmental exposure to asbestos. In areas such as Turkey where environmental exposure to asbestiform fibres is common, more than 50% of the population > 60 yrs of age may have pleural calcification [12] and mesothelioma may also develop. Asbestos-containing "white" soil is used as a whitewash or plastering material and is found in the home environment, resulting in a reversal of the traditional male predominance in asbestos-related disease. DPT may occur in up to 11% of this population [12] Studies from China [13], Finland [14] and Corsica [15] have all shown that pleural plaques are common, and one necropsy study reported plaques in 58% of cases of 288 urban men [16]. The prevalence of both DPT and pleural plaques increases significantly with past occupational exposure to asbestos, duration and intensity of exposure. Thus, workers who have worked in occupations with heavy exposure (e.g. laggers, insulators) are more likely to have pleural disease than those with moderate or minimal exposures [17].

Although there are few studies which have concentrated upon the natural history of the disorder, it seems likely that after the initial episode of pleural inflammation, which may be mild or severe and accompanied by a pleural effusion, the condition then plateaus[6]. In a minority of cases, recurring episodes of pleural inflammation may be the cause of further disease progression. Cessation of exposure is believed to slow progression, although information about this is limited[18].

## Aetiology

Although this review concentrates on asbestos-related DPT, this is a diagnosis of exclusion. The differential diagnosis includes tuberculosis, previous chest trauma especially haemothorax, previous surgery (e.g. coronary artery bypass grafting (CABG)), recurrent pleurisy e.g. due to repeated episodes of pneumonia, tuberculosis or rheumatoid arthritis, drugs (e.g. practolol, methysergide), fibrosing pleuritis, and post-radiotherapy (Table 1). A careful history should always be taken to exclude other causes, of which post CABG is increasingly common. No reliable figures are available to assess the relative proportion of asbestos-related rather than non-asbestos related causes but it is reasonable to assume that asbestos is responsible for the majority of the cases where there is a documented history of asbestos exposure and the characteristic radiologic appearances are seen.

**Table 1:** Clinical differential diagnosis of asbestos related diffuse pleural thickening

**Diffuse pleural thickening due to acute pleuritis:**
Pneumonia
Tuberculosis
Empyema
Connective tissue disease
Drugs (eg. practolol, methysergide)
Fibrosing pleuritis
Post radiotherapy
Post-traumatic diffuse pleural thickening eg. haemothorax
Post-surgery (particularly coronary artery bypass grafting

**Other diagnoses that may resemble diffuse pleural thickening:**

Pleural plaques
Mesothelioma
Other pleural- based tumours

DPT can occur after exposure to all types of asbestos and its development is thought to be dose-related[17]. Where asbestos fibre load burdens have been performed in the different asbestos diseases, these are highest for asbestosis[11]. One autopsy study of asbestos fibres in 13 asbestos exposed cases of DPT found the proportion of shorter chrysotile fibres to be higher than longer amphibole fibres in parietal pleura when compared to the lung parenchyma[19]. Total asbestos fibre counts in the parietal pleura were significantly lower than in the lung parenchyma and no differences were found between asbestos counts in subpleural versus central areas of lung. Sebastien *et al *[20] also demonstrated that there was an increased frequency of short fibres and a decreased frequency of long fibres from lung to pleura and that the frequency of asbestos fibres in pleura compared to the parenchyma was low. He concluded that retention of asbestos fibres in the parietal pleura is related to fibre size and type, and that lung parenchymal retention is not a good indicator of pleural retention[20].

Where comparative asbestos fibre burdens have been calculated, fibre counts are highest for asbestosis followed by DPT, pleural plaques and then malignant mesothelioma.[11,20-22] In one study of 192 British naval dockyard workers (96 with DPT and 96 with pleural plaques) the average exposure ratings for DPT did not differ from those for pleural plaques[22]. However, further analysis suggested that patients with bilateral DPT had significantly more exposure than those with unilateral disease[22]. In one transmission electron microscopy (TEM) study of 44 workers from Wittenoom in Western Australia there was a median count of 207.5 uncoated fibres/gram (× 10^6^) per gram of dry tissue in 44 deceased workers with asbestosis compared to 134.6 uncoated fibres/gram (× 10^6^) in 53 with mesothelioma, 9.026 uncoated fibres/gram (× 10^6^) with lung cancer and 0.92 uncoated fibres/gram (× 10^6^) in the reference population[23]. There are no electron microscopy studies from Australia comparing asbestos counts in DPT with other asbestos-related diseases. It has been postulated that pleural plaques develop due to intermittent asbestos exposure which allows time for fibres to be cleared to the pleura. In contrast to this, asbestosis is believed to occur due to heavy and more continuous exposure which overwhelms the fibre clearance mechanisms[24].

## Pathogenesis

How asbestos fibres reach the pleural space and cause pleural fibrosis is the subject of ongoing debate. Asbestos fibres may be inhaled, ingested or absorbed through the skin[25]. Inhalation is by far the commonest route by which pathological consequences occur. The mechanism by which they reach the pleural space and cause a variety of different pathologies is controversial.

Fibres that are inhaled and pass through the conducting airways are deposited on the Type 1 alveolar epithelial cells that line the walls of the bronchiolar-alveolar duct bifurcations[26]. These phagocytic cells cause migration or "translocation" of fibres into the interstitium, where the larger fibres like amphiboles are retained [27,28]. This may in some patients induce a macrophage-induced alveolitis[27]. Alveolar epithelial cell injury damages the fibroblasts and myofibroblasts, causing them to produce increased extracellular matrix. This can result in fibrosis (asbestosis). The ability of the lung to clear the fibres becomes overwhelmed. The shorter asbestos fibres like chrysotile are then transported to the pleural surfaces by macrophages through the lymphatics, where they induce acute pleuritis, pleural effusion and fibrosis[24]. It has been postulated that fibres may also reach the pleural space via embolisation to the costal blood stream or by direct migration through the visceral pleura[29].

The mechanisms underlying why asbestos causes such a dense pleural fibrosis in DPT are gradually becoming understood. Injury caused by asbestos fibres induces subpleural fibroblasts and mesothelial cells to produce scar tissue [30] and collagen deposition, resulting in subpleural thickening. It is still unclear why asbestos fibres which reach the pleura induce differing pathologies in individual patients, but is likely to be due to several mechanical, biochemical or genetic events[3]. The response of the mesothelial cell to injury and the ability of it and the basement membrane to maintain their integrity is pivotal as to whether or not fibrosis occurs, and cytokines, growth factors and reactive oxygen species (ROS) are likely to play a role[30]. Recent evidence from studies into other causes of pleural fibrosis suggests that upregulation of genes for pro-fibrotic mediators such as transforming growth factor beta (TGF-β) are important in asbestos-induced fibrogenesis. TGF-β and other cytokines such as tumour necrosis factor alpha (TNF-α) then cause disordered fibrin turnover, with increased fibrin formation and decreased and fibrin dissolution, resulting in the formation of a fibrinous intrapleural matrix[31]. TGF-β is likely to be the most potent pro-fibrotic mediator, recruiting fibroblasts, and initiating matrix remodelling. In animal studies, intrapleural injections of TGF-β_2 _rapidly induce pleural fibrosis and pleural sclerosis, [32,33] with concomitant generation of reactive nitrogen and oxygen species (RNS and ROS), possibly acting via iron in asbestos fibres. These are cytotoxic and stimulate fibroblasts to synthesise extracellular matrix[34,35].

Another theory proposes that individual differences in the inflammatory response to asbestos determine whether pleural plaques or DPT develop. This is supported by several animal studies. One such study showed that after installation of intra-pleural asbestos, the presence of large numbers of pleural macrophages led to pleural plaque formation while their paucity resulted in DPT[36]. Despite historical theories, it seems unlikely that direct mechanical irritation by asbestos fibres is responsible for the inflammatory infiltrate seen with asbestos. Inflammatory change is not seen at the site of pleural plaques, suggesting that this traditional explanation (irritation by fibres in the visceral pleura on the overlying pleura) may be incorrect[3]. In DPT there is fusion of both pleural layers with loss of the submesothelial elastic tissue, suggesting that significant inflammation has already occurred[3,37].

On a clinical basis, there are several mechanisms by which diffuse pleural thickening has been postulated to develop: subsequent to benign asbestos-related pleural effusion, following recurrent bouts of acute pleuritis and/or extension of parenchymal fibrosis (asbestosis) to the visceral pleura[31].

Asbestos fibres can induce an acute exudative pleural effusion which may be symptomatic or asymptomatic. Approximately one third of these effusions may be eosinophilic[37]. Benign asbestos related pleural effusions may precipitate the development of DPT via a complex interaction of inflammatory cells and cytokines locally within the pleural cavity[30]. This could explain why approximately one third of cases of DPT are unilateral[2,38]. Asbestos fibres which are coated in iron (asbestos bodies) are rarely found in pleural fluid, but they may occasionally be seen in pleural tissue. However, they are frequently seen in the lung tissue adjacent to DPT[24]. The frequency of pleural effusions before the development of DPT has been reported to range between 31.4% and 37%[2]. In a study of 2,815 insulators > 30 years from the onset of asbestos exposure, 20 had a past history of benign pleural effusion and of these, diffuse pleural thickening with blunting of the costophrenic angle was detected in 16[39]. Pleural effusions may produce symptoms of an acute pleuritis (i.e. chest pain on exertion, fever, malaise and mild dyspnoea) or they may be asymptomatic. They generally resolve spontaneously and do not predict the development of malignant mesothelioma. The pleural thickening and fibrosis may increase with each subsequent episode of pleural effusion.

DPT may also develop due to recurrent episodes of asbestos-induced acute pleuritis in the absence of detectable pleural effusion. Here, a fibrinous matrix is laid down, matures and organizes into dense collagenous material [3,30,31]. However, this may merely represent a milder degree of the same pleural inflammation responsible for recurrent effusions. This is difficult to confirm because serial chest radiology is not usually performed without clinical indication. Another theory as to the pathogenesis of diffuse pleural thickening is that it is an extension of the parenchymal fibrotic process to the visceral and parietal surface causing inflammation and fibrosis to the superficial or visceral pleural lymphatics[29]. However DPT and asbestosis are said to occur together in only 10.3% of cases and such a suggestion therefore does not account for the remaining 90% of cases [2].

Pleural plaques and DPT frequently coexist. However, they differ in their site of origin, appearance, extent, symptomatology, functional impairment and prognosis. Pleural plaques are discrete areas of relatively acellular and avascular pleural fibrosis that arise from the parietal pleura and the superior surface of the diaphragm[3]. The most widely accepted theory for the development of pleural plaques is that the asbestos fibres travel via retrograde lymphatic drainage from the mediastinal lymph nodes to the retrosternal and intercostal lymphatics and thence to the pleural space[31]. Another less plausible explanation is that fibres protruding into the pleural space cause local inflammation to the parietal pleural surface. Pleural plaques differ from diffuse pleural thickening in a number of ways. Unlike DPT, pleural plaques are sharply demarcated from surrounding structures. They have a prolonged latency of at least 10 to ≥ 40 years and it is controversial whether they produce symptoms and functional impairment unlike diffuse pleural thickening. Some studies have shown that presence of pleural plaques may result in reductions of FVC but not the FEV_1_/FVC ratio[11]. They are frequently incidentally detected on chest radiography and they are a helpful marker of previous asbestos exposure. Their presence is associated with a higher risk of malignant mesothelioma and lung cancer compared with workers with a similar exposure history but no plaques [40], but there is no evidence to suggest that they are in themselves pre-malignant.

## Macroscopic appearance

The lungs in DPT are surrounded by grey fibrous tissue, which blends with surrounding normal pleura. Unlike pleural plaques, DPT is not sharply demarcated and is often associated with fibrous strands ("crows feet") and parenchymal bands that extend into the lung parenchyma and lobular septae[11]. These do not however represent asbestosis. Occasionally, pleural plaques may be superimposed on DPT and may also occur separately within the thoracic cavity. DPT is more extensive than pleural plaques. It may be unilateral or bilateral, and in contradistinction to pleural plaques it arises from the visceral not parietal pleura. DPT often results in dense adherence between parietal and visceral pleural layers. It may encase the lungs and obliterate pleural spaces, lobar fissures and the costophrenic recesses[6].

Macroscopically, pleural plaques have a white or pale yellow shaggy "candle wax" appearance, very different from DPT. Microscopically they consist of acellular interwoven bundles of collagen[3]. Central calcification may occur in mature lesions usually > 30 years old.

## Clinical findings

DPT has been reported as associated with a number of symptoms (Table 2). It is suggested that both DPT and pleural plaques are independently associated with exertional dyspnoea. One study looking at a selected series of compensated patients with moderate to severe DPT found that 95.5% complained of breathlessness 65% of moderate breathlessness and 11% of severe breathlessness [6]. A single case report has found diffuse pleural thickening to be associated with hypercapnoeic respiratory failure due severe restrictive lung disease and death[41].

**Table 2:** Clinical characteristics of asbestos-related diffuse pleural thickening

Prevalence	5–13.5% of asbestos exposed people 3–34 years following first asbestos contact
Latency	Variable but can occur within 1 year of a benign asbestos associated pleural effusion. Usually 15–20 years
Frequency	Increases from the time of first exposure
Pathogenesis	Uncertain. Possible sequela of benign asbestos associated pleural effusion, recurrent bouts of asbestos related pleuritis or extension of parenchymal fibrosis into the pleura
Location	Usually bilateral, 1/3^rd ^are unilateral Can extend to encase the lung, obliterating the pleural spaces, the fissures and the costophrenic recesses
Macroscopic appearance	Arises from the visceral pleura. Pale grey diffuse thickening of visceral pleura that may become adherent to the parietal pleura. Not sharply demarcated from the pleura, unlike pleural plaques.
Microscopic appearance	Collagenous fibrous tissue
Symptomatology	Chest pain, dyspnea. Hypercapnic respiratory failure and death in severe cases
Pulmonary function	Restrictive defect. Reduction in static lung volumes and compliance. Reduced transfer coefficient (TLCO) but a raised or maintained TLCO when corrected for alveolar volume (KCO)
Chest x-ray appearance	Smooth non interrupted pleural density extending over at least 1/4^th ^of the chest wall Obliterates the costophrenic angles
HRCT appearance	A continuous sheet of pleural thickening more than 5 cm wide, more than 8 cm in craniocaudal extent and more than 3 mm thick
Associated features	Rounded atelectasis, parenchymal bands
Treatment	Supportive, symptomatic, non invasive ventilation for respiratory failure
Differential diagnosis	Any cause of acute pleuritis can cause diffuse pleural thickening (see table 1). Chest trauma and surgery, Mesothelioma, other pleural based tumours, pleural plaques.

Chronic chest pain may also be a feature of DPT, although this is usually mild. Mild to moderate chest pain was noted in over half of the patients with moderate to severe DPT in a study of more severe cases[6]. This was more frequent than in previous studies, probably because of the selected population. The pain is generally described as dull in character. In another study, patients with benign asbestos-related pleural and parenchymal disease appeared to have higher rates of chest pain, particularly anginal chest pain as assessed by a cardiovascular survey questionnaire[42]. More severe pain seemed to be experienced in those with heavier asbestos exposure, older subjects and in retired workers. However, it is not clear if the incidence of ischaemic heart disease is truly higher in asbestos-exposed workers or if asbestos-related lung disease merely causes pain that resembles angina[42]. The presence of radiographic pleural thickening has been shown to be a risk factor for death from ischaemic heart disease in subjects exposed to crocidolite from the Wittenoom population[43]. One Swedish study reported a higher age and gender associated prevalence of calcified pleural plaques in patients with coronary artery disease (35%) compared with those with lung cancer (19%)[44]. The relative risk adjusted for age and gender was 2.19 (95% CI 1.44–3.32) among patients referred consecutively for coronary angiography compared with lung cancer patients. For this group, however, calcified pleural plaques showed no association with the severity of coronary artery disease, diabetes, hyperlipidaemia or smoking. It is unclear whether these results are due to confounding factors or whether there is a true aetiological association.

## Pulmonary function

DPT may be associated with a "constrictive" deficit in pulmonary function. [6] A reduction in static lung volumes and lung compliance with reduced transfer coefficient (TLCO or DLCO) and a raised or maintained transfer coefficient (KCO) occurs[6]. This restriction may occur independently of the presence of asbestosis. The extent of DPT is strongly correlated with decreasing lung volumes, especially with residual volume, and less strongly with increasing transfer coefficient or KCO. Few longitudinal studies exist, but these have found no correlation between radiographic severity and longitudinal loss of lung function [6,45].

It has been suggested that restriction in DPT is due to adhesion of the parietal and diaphragmatic pleura in the zone of apposition between the diaphragm to the chest wall [46]. This limits separation of the diaphragm from the rib cage during inspiration, which reduces the volume contributed by motion of the diaphragm and lower rib cage. It is thought that it is the reduction in movement of the lower rib cage that is the major cause of restriction, because the reduction in volume contributed by the diaphragm is partly compensated by flattening of its dome. Five HRCT scoring systems to measure the area and thickness of abnormal pleura have been reviewed by Copley et al[45]. The extent of DPT on HRCT is strongly correlated with decreasing FVC and TLC and less strongly with increasing transfer coefficient KCO. However, in some patients the decreased DLCO suggests that pulmonary fibrosis may have been contributing to restriction[3].

The natural history of DPT is probably benign but this is difficult to assess as relevant studies either include a large proportion of cases of pleural plaques or concentrate on selected populations. In the longitudinal study of Yates et al [3], the pattern of lung function change was of an initial large loss of lung function followed by relative stability. There was further loss of lung function in a minority of cases[6].

## Imaging

DPT is most commonly assessed by the plain chest radiograph, although CT scanning is increasingly superseding this tool The chest radiographic appearance is of a continuous, irregular pleural shadowing which may extend up both chest walls and blunt one or more costophrenic angles[3] (Figure 1). This density should extend over at least one quarter of the chest wall. The revised 2003 International Labour Office (ILO) Classification of Radiographs of Pneumoconioses provides a system for classifying pleural plaques and diffuse pleural thickening and for differentiating between these disorder[47]. DPT is recorded only in the presence of, and in continuity with, an obliterated costophrenic angle. The earlier 1980 ILO version did not require obliteration of the costophrenic angle, which is now required. However, the chest radiograph even when accompanied by an oblique film is an insensitive index of disease severity[6].

It is well established that high resolution CT scanning is more sensitive and specific than chest radiography for the diagnosis of DPT, pleural plaques and asbestosis[5]. It can detect early pleural thickening (ie 1–2 mm in thickness), and several classification systems have been devised[17,45,48-50]. The most commonly used in Australia is that of Lynch *et al*.[49] Here, diffuse pleural thickening is defined on HRCT as a contiguous sheet of pleural thickening more than 5 cm wide on transverse CT images, more than 8 cm in extent in craniocaudal images and more than 3 mm thick[49,51] (Figure 2). Pleural calcification rarely occurs in DPT, unlike pleural plaques (Figure 3). HRCT should ideally be performed with prone views and at full inspiration to avoid dependant atelectasis in the posterior lung fields which may be confused with parenchymal fibrosis[11]. A rare variant of apical diffuse pleural thickening in association with apical fibrosis has also been reported [52].

The correlation of CT abnormality with symptoms has not been well investigated. Five methods of quantifying pleural thickening were compared by Copley et al in 50 patients with benign asbestos-related disorders. Comparable functional-morphological correlations were achieved by the different systems but the subjective simple CT system was easy to apply and useful for accurate assessment of the lung parenchyma [45]. HRCT is also a sensitive method for assessing plaques, and is more specific than chest radiography for distinguishing DPT from other structures such as extrapleural fat [11].

Magnetic resonance imaging (MRI) and fluorodeoxyglucose (FDG)- positron emission tomography (PET) imaging scan may be useful in distinguishing malignant from benign pleural disease.[53-57]. Two studies of patients with pleural disease suggested that when signal intensity and morphologic features are assessed, MRI is superior to CT in differentiating benign and malignant pleural disease with a sensitivity ranging from 98–100% and a specificity of 92–93% [53,54]. High signal intensity in relation to intercostal muscles on T2-weighted and/or contrast enhanced T1-weighted images was significantly suggestive of malignant disease. One study also suggested that MRI has a higher interobserver agreement compared with CT in detecting pleural thickening, pleural effusion and extrapleural fat [56]. The agreement was however similar for the detection of pleural plaques and CT was superior for the detection of pleural calcification which is a marker of benign disease.

PET can help distinguish between malignant pleural mesothelioma which has higher glucose avidity and benign DPT in patients where the two diagnoses coexist. A prospective American study of 28 patients referred for the evaluation of suspected mesothelioma demonstrated that a standardized uptake value for FDG of 2.0 used to differentiate between malignant and benign disease had a sensitivity of 91% and a specificity of 100%[55]. However some epithelial mesotheliomas had a glucose avidity that is very close to this threshold of 2.0 [55]. Another study of 63 patients with mesothelioma suggested that while PET did not identify the local extent of tumour or mediastinal nodal metastases it does detect extrathoracic metastases reducing the need for inappropriate thoracotomy[58]. Moreover, one recent study has suggested that PET can predict survival in mesothelioma[59]. PET needs to be used in conjunction with an anatomic imaging study like CT [60] when staging mesothelioma. The cost and availability of MRI and PET are factors that may limit their use in some centres when compared with CT.

## Associated features

Several other features are frequently associated with DPT on HRCT in addition to pleural plaques. These include parenchymal bands and rounded atelectasis[11]. Parenchymal bands are linear 2–5 cm long opacities extending through the lung to make contact with the pleura[11]. These bands are areas of fibrosis along bronchovascular sheaths or interlobular septa and are generally related to moderate pleural fibrosis[61]. Small pleuro-parenchymal bands known as "crow's feet" are associated with focal rather that diffuse pleural thickening [22]. The radiological appearances are different from asbestosis. Gevenois and colleagues distinguish between these features and those secondary to asbestosis, where septal and interlobular lines and honeycombing may be seen [61].

**Figure 4:**
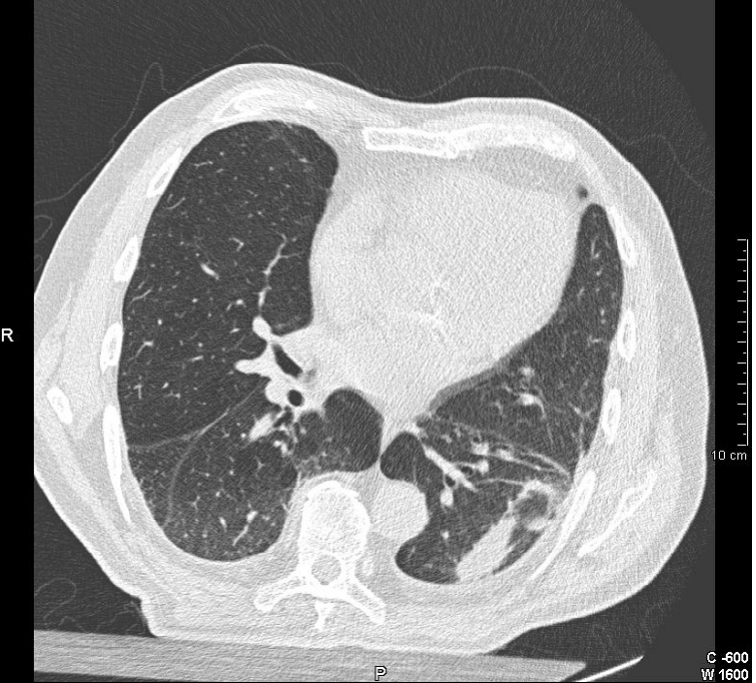
CT scan of the thorax demonstrating "folded lung" or Blesovsky's syndrome in association with diffuse pleural thickening.

Rounded atelectasis may also occur in association with DPT. It is known as shrinking or contracted pleuritis, a pleuroma or "Blesovsky"s syndrome" (Figure 4)[62]. Rounded atelectasis is believed to be the result of infolding of the thickened fibrotic visceral pleura with collapse and chronic inflammation of the underlying lung parenchyma. The "comet sign", or a rounded mass connected by a fibrous band to an area of thickened pleura, is the pathognomonic HRCT feature. It can occur in response to any cause of acute pleuritis, but asbestos appears to be the commonest recognised cause[11]. Symptoms generally only occur if the area of atelectasis is large enough to compromise lung function[3]. The differential diagnosis of rounded atelectasis includes a peripheral lung cancer or a benign inflammatory pseudotumour. The latter, however, generally evolves more quickly[11].

## Differential diagnosis

There are several important differential diagnoses for asbestos-related DPT (Table 1). Any cause of acute pleuritis can cause pleural thickening which is clinically indistinguishable from that due to asbestos. Examples of these include tuberculosis, previous trauma, empyema, connective tissue diseases, drugs and surgery [11,31] including coronary artery bypass surgery (CABG) [63]. These are much more likely to be unilateral, whereas although DPT can occur unilaterally, this is less common. Calcification may be heavy in post-tuberculous pleural thickening or that occurring after haemothorax. With past tuberculosis, upper lobe fibrosis may also occur along with bronchiectasis and evidence of old surgical procedures e.g. thoracoplasty. Upper lobe pleural thickening, especially when bilateral, is more likely to be due to old tuberculosis in older patients; however, upper lobe disease has also been described after asbestos exposure (46) and there are no radiological features which are 100% specific. Clinical correlation is required, and a good occupational history is invaluable.

Because of the wide differential diagnosis for DPT, it is less specific for asbestos exposure than pleural plaques [18,64]. The most important diseases which need to be distinguished from DPT and asbestos related chronic pleuritis and effusion are malignant mesothelioma, and of course tuberculosis in areas of high prevalence. Empyema should also be also included, although features of sepsis are usually suggestive and the history more acute. Mesothelioma is unlikely when the chest pain is mild and persists for years with minimal or no clinical or radiographic progression. CT and MRI features which are more frequent in malignant disease include circumferential pleural thickening (> 1 cm) with nodularity and irregularity of pleural contour as well as infiltration of the chest wall or diaphragm and mediastinal, pleural and/or nodal involvement[53,65]. Involvement of the mediastinal pleura is thought to be more suggestive of mesothelioma than DPT. As discussed above, MRI and PET maybe useful to distinguish malignant and benign pleural disease[53-56]. Tuberculosis is more likely to be associated with fever, lymphocytosis and a history of haemoptysis. Differentiating between the different causes of pleural disease can be very difficult and should include all possible information including that from pleural aspiration and/or cytology and biopsy where appropriate.

## Management

Few data exist relating to optimal management of patients with DPT, probably because the condition is uncommon on its own. The majority of patients have not been shown to require ongoing respiratory specialist management and are treated symptomatically in primary care. The optimal management of more severe cases has not been well studied. Pulmonary rehabilitation has not been investigated in this area, and conventional respiratory therapies are likely to be ineffective other than analgesia where needed. There are reports of non-invasive ventilation being used to support patients with respiratory failure due to diffuse pleural thickening [66], but this is rarely required unless other pathologies exist. It has also been suggested in the past that patients with DPT might benefit from decortication because they have increased elastic recoil and a normal diffusing capacity when corrected for alveolar volume, but surgery has been rarely applied [67]. However, patients with asbestos-related disease often have other co-morbidities which preclude surgery, and surgical treatment is unlikely to be appropriate if clinically significant asbestosis is present. Significant pleural disease is associated with a higher rate of post-operative complications and therefore most surgeons are reluctant to embark on such a procedure. However, surgery may be highly effective for patients with pleural disease due to other causes. This has not, however, been formally studied for asbestos-related DPT.

## Conclusion

DPT is now a well recognised consequence of asbestos exposure and benign asbestos related pleural effusions, although it is probably under- recognised and reported. It may cause dyspnoea, chest pain, respiratory failure and a "constrictive" pattern on pulmonary function testing, but is usually only mildly symptomatic. It has distinctive features macroscopically, histologically and on HRCT. DPT may coexist with pleural plaques and has a distinctly different pathology with a different natural history, radiology and prognosis. Treatment is largely limited to supportive and symptomatic care. The incidence of this disease is currently rising and total numbers are likely to exceed those of malignant mesothelioma in the future. Thus, more clinicians are likely to be involved in its management and further research is required to better elucidate its natural history, radiology and treatment.

## Competing interests

The authors declare that they have no competing interests.

## Authors' contributions

DY conceived the manuscript, SM drafted it, and AS, DY and AJ edited it and contributed additions to the text. All authors read and approved the final manuscript.
